# Genomic profiling for non-small cell lung cancer: Clinical relevance in staging and prognosis

**DOI:** 10.1097/MD.0000000000036003

**Published:** 2023-11-24

**Authors:** Abhinav Bhattarai, Sangam Shah, Hashem Abu Serhan, Ranjit Sah, Sanjit Sah

**Affiliations:** a Institute of Medicine, Tribhuvan University, Kathmandu, Nepal; b Hamad Medical Corporation, Doha, Qatar; c Department of Microbiology, Tribhuvan University Teaching Hospital, Institute of Medicine, Kathmandu, Nepal; d Department of Microbiology, Dr. D. Y. Patil Medical College, Hospital and Research Centre, Dr. D. Y. Patil Vidyapeeth, Pune, Maharashtra, India; e Datta Meghe Institute of Higher Education and Research, Jawaharlal Nehru Medical College, Wardha, India; f Research Scientist, Global Consortium for Public Health and Research, Datta Meghe Institute of Higher Education and Research, Jawaharlal Nehru Medical College, Wardha, India; g SR Sanjeevani Hospital, Siraha, Nepal.

**Keywords:** chemotherapy, NSCLC, profiling, prognosis, risk

## Abstract

Lung cancer is one of the most common cancers prevalent and around 80% of all cases are non-small cell lung cancer (NSCLC). Due to high recurrence rates, the mortality of NSCLC is high. Conventional staging systems allowed risk classification of patients in order to simplify the patient selection for adjuvant chemotherapy. Gene expression analysis has been shown to possess advantage over conventional staging systems in NSCLC in terms of patients risk classification. This article reviews the evidences on the genomic profiling of NSCLC patients into high and low-risk groups based on the expression of genes involved in various proliferative pathways.

## 1. Introduction

The capability of genomic testing is growing to provide greater benefits for patients and healthcare systems in the context of personalized medicine, which is currently undergoing change. By leveraging extensive and carefully maintained genetic databases and generating evidence to guide testing methodologies, treatment alternatives, and associated clinical decision support, increased genomic testing deployment can enhance clinical value. The use of genomic mutational-methylation signatures in cancers has been widespread for the past decade. While multi-gene assays have increasingly received FDA approval and are now incorporated into cancer screening protocols, several other signatures are undergoing trials and have shown promising results. Currently, BreastNext, MammaPrint, and Oncotype DX have been widely employed in the risk characterization of breast cancers.^[1–3]^ As such, ConfirmMDx, ExoDx for prostate cancer^[4,5]^ and Epi proColon, ColoVantage for colorectal cancer have been widely recognized genomic signatures.^[6,7]^ Additionally, these signatures have significantly aided in therapeutic indication, determining prognostic trajectory, and importantly, making decisions.

Lung cancer has been recognized as one of the commonest cancers prevalent today. In 2020, lung cancer was ranked second position on the list of worldwide cancer prevalence with 2.2 million new cases and the numbers have been outrageously skyrocketing. Lung cancer is the most prevalent cancer type in men and the second most prevalent cancer after breast cancer in women. Approximately, 12.5% of all cancer patients have lung cancer. Despite the development of advanced diagnostic tools and treatment options, lung cancer still is the leading cause of cancer deaths.^[8]^

Non-small cell lung cancer (NSCLC) is a more frequent subtype that comprises around 80% of all lung cancer cases and is known for its poor prognosis and high mortality rate in patients. Different types of NSCLC: adenocarcinoma, squamous cell carcinoma, and large cell carcinoma have been identified.^[9]^ These tumors have a disastrously high recurrence rate ranging from 35% to 50%, making them one of the most difficult cancers to treat. Despite surgical resection of the tumor, a 5-year mortality rate of 30% to 50% in patients diagnosed with Stage I–IIA NSCLC has been reported and in the last 30 years, there has been very minimal progress in patient survival. This high mortality has been attributed to widespread distant metastases and recurrence. Interestingly, research has found that the timely administration of adjuvant duplet cisplatin-based chemotherapy can enhance the 5-year survival rates by 4% to 15% in these patients.^[10,11]^ It is therefore paramount to identify the patients who fall within this 30% to 50% category of high risk of recurrence and death to impart appropriate treatment selection and minimize lung cancer morbidity and mortality.

Attempts for identifying high-risk NSCLC patients were made in 1953 by the proposal of the conventional TNM (Tumor Node Metastasis) system and currently, the 8 edition of the conventional TNM staging scheme is adopted.^[12,13]^ However, external studies performed to validate the risk classification of this system has yielded unsatisfactory findings and low discriminatory ability.^[12,14–16]^ Currently, emphasis has been rising on the genome-based screening of NSCLC patients and results have been promising for the precise discrimination of high risk patients. Studies have advocated the benefits of the genomic screening on the treatment and overall survival of NSCLC patients, which led to the formulation of standardized genomic profiling assays.^[11,12,17,18]^ This article has aims to reflect the evidences and analyze the utility of a standardized genomic profiling assay which consists of 14 tumorigenic genes potentially resulting in NSCLC.

## 2. Development of genetic signatures in NSCLC

Considering the inadequacy of the TNM system for NSCLC staging and prognosis, continuous attempts has been made utilizing genomic profile to achieve accurate treatment selection. Earlier researchers studied numerous gene involved in different proliferative pathways via microarray technique and found supportive evidences. Later, most studies utilized polymerase chain reaction (PCR) in a quantitative setting used to detect complementary DNA by reverse transcription of the target gene transcripts.^[19]^ Model establishment was a crucial aspect since gene and mutations responsible for proliferative pathways often overlap in a variety of cancers and the selection of least/nonoverlapping genes for NSCLC was challenging. These studies further narrowed down the list via selection of the topmost genes in the hierarchy after robust studies on snap frozen tissues of non-small cell lung tumors. A brief review on the emergence of genomic profiling in lung cancer has been performed by Kratz et al^[20]^ Addition of such molecular signatures has been shown to supplement the discriminatory ability of the eighth edition of TNM staging criteria. In this regards, the TNMB (B for biological) was proposed which utilized a panel of 14 signature genes as a risk classifier in NSCLC.^[12]^ The 14 gene genomic profiling has been gaining popularity and its application has been emerging in lung cancer personalized treatment. Simultaneously, investigations on the 14 gene genomic profiling has been rising and promising results has been documented. Table 1 displays the signature genes used in the profile and the relevant tumorigenic pathways involved are summarized.

**Table 1:** Genes screened in the DetermaRx assay.

S.N.	Signature genes	Gene name	Protein function
1.	*BRCA1*	Breast cancer 1, early onset	Tumor suppression; mend DNA breaks
2.	*CDC6*	Cell division cycle 6 homologue	DNA replication; formation of pre-replication complex
3.	*CDK2AP1*	Cyclin-dependent kinase 2 associated protein 1	Regulation of DNA replication; negative regulation of CDK activity in S phase; epigenetic regulation via nucleosome remodeling
4.	*ERBB3*	V-erb-b2 erythroblastic leukaemia viral oncogene homologue 3	Formation of epidermal growth factor receptor (EGFR) and promote cell proliferation via tyrosine kinase activity.
5.	*FUT3*	Fucosyltransferase 3	Tissue differentiation, and tumor metastasis
6.	*IL11*	Interleukin 11	Transmembrane signaling and cellular proliferation
7.	*LCK*	Lymphocyte-specific protein tyrosine kinase	Cell signaling and T-cell maturation
8.	*RND3*	Rho family GTPase 3	Signal transduction via GTPase channel
9.	*SH3BGR*	SH3 domain binding glutamic acid-rich protein	Chemical signal transmission via Wnt, regulation of cellular proliferation
10.	*WNT3A*	Wingless-type MMTV integration site family, member 3A	Regulation of cell fate
11.	*ESD*	Esterase D	Recycle of sialic acid
12.	*TBP*	TATA box binding protein	Regulation of transcription; recognition of promoter sequence
13.	*YAP1*	Yes-associated protein 1	Regulation of cellular growth, development, and repair via Hippo signaling pathway.
14.	*BAG1*	BCL2-associated athanogene	Apoptosis

## 3. Standardized genomic profile and assay performance

Recently, a commercially available 14-gene PCR assay called the DetermaRx has been developed by Oncocyte® which quantitates the expression of genes that are found to be abnormally expressed in the case of NSCLC.^[21]^ Based on the gene expression data, risk scores are generated which are further stratified to categorize the patients into low, intermediate, and high-risk groups which correlate with the risk of recurrence and 5-year mortality.^[12]^ Furthermore, the DetermaIO has been implicated in predicting response to immunotherapy.

The analytical validation of this assay has been performed by various external studies and has yielded high sensitivity and specificity. This assay is easy to perform and utilizes formalin-fixed paraffin-embedded tissue, excised from the tumor site. The assay doesn’t require additional biopsies to be performed. It is performed from the tissue that is surgically resected from the lung. The same tissue can be used for multiple testing including histopathology, immunohistochemistry, and molecular profiling. The mRNA is then extracted from FFPE and pre-amplification is allowed using gene-specific primers. Reverse transcription is carried out to form complementary DNA which is then amplified using the standard TaqMan PCR amplification. The gene expression is then calculated relative to the commercially available RNA extracted from frozen normal lung samples. The risk scores range from 1 to 100. A lower score is interpreted as a low risk of mortality within 5 years of resection, whereas, higher scores indicate a high chance of mortality within 5 years and an elevated risk of recurrence.^[18]^ Categorization as “high-risk” necessitates the use of adjuvant chemotherapy whereas in the “low-risk” category, the chemotherapy is not indicated. This way, low-risk patients need not suffer the toxic effects and financial burden of the chemotherapy and in high-risk patients, recurrence can be avoided and mortality can be reduced significantly.

**Figure 1.**
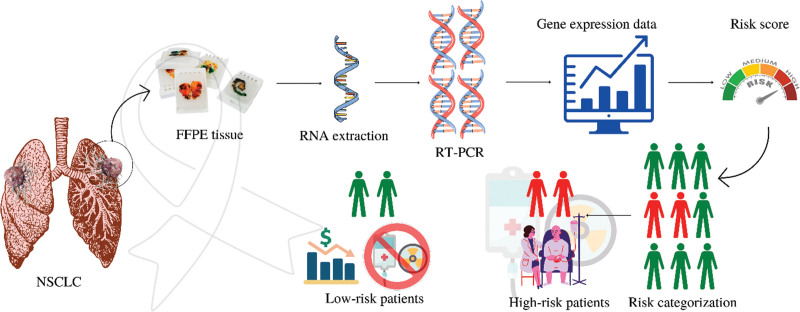
Genomic profiling in NSCLC: procedure and implications.

The overall procedure and its implications are summarized in Figure 1.

## 4. Evidences so far

The results from randomized trials performed in US validation cohorts have found that adjuvant chemotherapy was actually unnecessary for the low-risk group. Results showed that only 5% of NSCLC patients categorized into the low-risk group based on the genomic profiling, developed recurrence and these patients have a 94% survival rate without adjuvant chemotherapy. The adjuvant chemotherapy was therefore implemented only in the high-risk group which could successfully produce a cancer-free status in a significant proportion of the patients.^[22]^ EGFR Tyrosine Kinase Inhibitors (TKIs) are commonly used adjuvant immunotherapy for patients resistant to chemotherapy and its efficacy in patients harboring EGFR mutations has been extensively studied. The 14-gene testing has shown advantage in the case the molecular high-risk EGFR-mutant NSCLC patients too and has contributed in accurately identifying the target population, and the resultant reallocation of population previously receiving inadequate EGFR-TKI into molecular-high-risk have subsequently shown survival benefit.^[23]^ The FLAURA trial showed similar results on the utility of EGFR mutation profiling using *cobas* for patient selection and treatment with Osimertinib, on such EGFR TKI.^[24]^

Notably, this genomic profiling has been clinically validated in blinded clinical trials involving 1500 participants and have found that it could predict the risk of mortality in the high-risk group with more accuracy than the National Comprehensive Cancer Network (NCCN)’s clinico-pathological criteria.^[17,25]^ A study compared the incidence of recurrence in patients classified into low and high risk groups based on NCCN and TNMB (B for biology) criteria which utilizes the 14-gene panel and found that while both low and high risk NCCN groups had recurrences, only the high risk patients based on the TNMB classification had recurrence.^[26]^ This justified that the genomic profiling is more accurate in the risk categorization of NSCLC patients as compared to other schemes.

The improvement in the clinical decision making by the utilization of the test results has been evident by a study that investigated the impact of the genetic testing on physicians’ treatment decisions.^[27]^ The study compared the changes in chemotherapy treatment recommendation of 58 physicians, pre and post testing. The investigators reported a 30.8% change in the treatment recommendation to the patient after the testing. Among patients who were not recommended chemotherapy prior to testing, 34.6% of them were recommended for treatment after the results were obtained. The study further found that patients belonging to the high-risk group had the most changes in treatment recommendations (44.4%) post testing. The genetic risk categorization wasn’t only effective within the gray zone between stage II and III, but also showed efficacy in early-stage. The study also found that 32.5% of stage I NSCLC patients who were not recommended chemotherapy, received recommendation after the test results. Likewise, in 20.6% of patients who were initially recommended for chemotherapy, their recommendations were withdrawn based on the test results. In the United States, the assay has resulted in an average saving of $11,608 per patient via obliterating the unnecessary use of chemotherapy in low-risk patients and wiping out the requirement for recurrence testing in high-risk patients subjected to prior adjuvant chemotherapy and immunotherapy.^[28]^

Other studies too have supported that the molecular testing has aided in patient treatment decisions and prognosis superior to other conventional criteria. Woodard et al’s study compared the 5-year disease-free survival (DFS) estimates of NSCLC patients based on the NCCN and molecular testing criteria.^[11]^ The estimate DFS based on the NCCN’s criteria was 75.2% for untreated lower-risk patients and 61.9% for untreated higher-risk patients. In contrast, the molecular classification estimated a 93.8% 5-year DFS for untreated low-risk patients and a 48.9% DFS for untreated higher-risk patients. Seemingly, the molecular test categorization scheme showed a greater discriminative strength in classifying patients for chemotherapy recommendations. When the untreated higher-risk patients were then administered chemotherapy, the DFS was estimated to increase up to 91.7% which can be considered a breakthrough in NSCLC treatment recommendations. However, these are only estimates and the use of superlatives in oncology without justifiable evidences are indeed misleading.^[29]^

As a solid ground evidence, Kratz et al^[12]^ in 2019 presented their report on the comparison of the conventional NSCLC staging schemes and the novel molecular prognostic classifier. The net reclassification improvement between the 6th versus seventh and the 7th versus 8 TNM editions were not significant (–0.01 and 0.03 respectively). In contrast, there was a significant increase in the net reclassification improvement between the 8 edition of TNM classification and the TNMB molecular classification (0.33). The investigators also performed the Kaplan–Meier survival analysis and found a superior survival curve separation when the molecular classification was used for the risk categorization. Another study^[30]^ reported significant differences in the survival of the patients between high, intermediate, and low risk categories (52.3%, 69.1%, and 83.0%, respectively). A high-risk categorization significantly predicted morality on a cox hazard analysis with a hazard ratio of 3.31. These findings provide a strong statistical evidence of the ability of the genomic profiling in the accurate and comprehensive risk categorization of NSCLC patients so as to impart precise treatment recommendations and mitigate the bearing of chemotherapeutic toxicities in low-risk patients.

Exon 19 deletions (19 Del) and exon 21 codon p.Leu858Arg (L858R) point mutations of the epidermal growth factor receptor have been shown to be significant predictors of response to EGFR tyrosine kinase inhibitors in NSCLC.

## 5. Conclusion

Genomic profiling for NSCLC has shown impressive results in clinical decision making, and improving patient outcomes. Additionally, it can ensure optimum mobilization of health finance by obliterating the unnecessary administration of chemotherapy to low-risk patients. On the other hand, application of adjuvant chemotherapy to the high-risk individuals has shown efficiency in enhancing the 5-years overall survival and disease free survival rates. Incorporation of genomic profile when dealing with NSCLC can significantly aid in imparting personalized treatment and prove to be a breakthrough in precision oncology.

## Author contributions

**Conceptualization:** Abhinav Bhattarai, Sangam Shah.

**Methodology:** Sangam Shah.

**Supervision:** Hashem Abu Serhan, Ranjit Sah, Sanjit Sah.

**Validation:** Sanjit Sah.

**Writing – original draft:** Abhinav Bhattarai, Sangam Shah, Ranjit Sah.

**Writing – review & editing:** Abhinav Bhattarai, Sangam Shah, Hashem Abu Serhan, Ranjit Sah, Sanjit Sah.
